# Standing by or Standing up? Victim Ethnicity, Bystander Responses and Psychological Distress in Different Types of Bullying Situations

**DOI:** 10.3390/healthcare14142194

**Published:** 2026-07-20

**Authors:** Emilia Alexandra Pascal, Octav Sorin Candel, Oara Prundeanu

**Affiliations:** Faculty of Psychology and Educational Sciences, Alexandru Ioan Cuza University of Iași, 700554 Iași, Romania; octav.candel@uaic.ro (O.S.C.); oara.prundeanu@uaic.ro (O.P.)

**Keywords:** bullying, cyberbullying, indirect bullying, bystander responses, victim ethnicity, psychological distress

## Abstract

**Highlights:**

**What are the main findings?**

- Bullying type shaped participants’ appraisals and bystander reactions
- Psychological distress was related to previous bullying experiences (as victim, bystander and aggressor) as well as hypothetical passive bystander reactions

**What are the implications of the main findings?**

- Anti-bullying interventions should address each form of bullying differently
- Indirect, verbal and cyberbullying should be especially targeted and interventions should aim to reduce their normalization and decrease passive bystanding

**Abstract:**

Background: Bullying represents a complex problem affecting students of all ages. Among the factors that influence it, appraisals of the transgressions and bystanders’ responses play a very important role. The present study examined adults’ emotional, evaluative, and behavioral responses to hypothetical bullying scenarios varying by bullying type and victim ethnicity. It also explored whether psychological distress was associated with bystanding behaviors as well as previous bullying experiences. Methods: The study used a mixed 3 × 4 vignette-based design. Victim ethnicity (i.e., Romanian, Roma, or Hungarian) was manipulated between participants, while bullying type was manipulated within participants through four scenarios describing physical, verbal, indirect, and cyberbullying. The final sample included 477 adults aged between 18 and 52 years. Participants appraised each transgression (i.e., empathic reactions, unacceptability), reported their likely bystander behavioral intentions (i.e., defend, ignore, report or join the aggression), completed the Depression Anxiety and Stress Scale (DASS-21) and reported previous bullying experiences as victim, witness, and aggressor. Results: Bullying type was the most consistent factor shaping participants’ appraisals as well as bystander reactions. The most tolerated forms were indirect bullying (e.g., highest probability to join the aggression) and verbal bullying (e.g., lowest unacceptability) the least accepted was physical bullying (e.g., lowest probability to join the aggression) while cyberbullying elicited specific patterns (i.e., lower empathy but higher willingness to report the incident). Victim ethnicity had a very limited effect, only concerning reporting intentions: lower for Hungarian targets compared to Romanian. Psychological distress was mainly associated with previous victimization and witnessing, while associations with scenario-based responses were smaller and more specific. Conclusions: The findings suggest that bystander responses are differentiated across bullying forms and prevention efforts should address subtle, indirect, verbal, and online forms of bullying, as these may be more easily normalized or less directly confronted.

## 1. Introduction

Bullying remains a widespread problem in educational contexts across the world, with international reports indicating that a substantial proportion of students experience at least one form of victimization [1,2]. Usually defined as a repeated and intentional aggressive behavior involving a real or perceived imbalance of power [3] bullying can have many forms and is generally associated with increased distress when experienced as a victim, witness or perpetrator [4,5,6]. However, intervention in bullying situations remains difficult, as the appraisal of such incidents is influenced by a range of factors, including the characteristics of the victim [7].

Traditional bullying can be differentiated into direct and indirect forms. Physical bullying refers to direct bodily aggression, such as pushing, hitting, tripping, or other behaviors involving physical force. Verbal bullying is also a direct form of aggression, but it involves words rather than physical contact, such as insults, mocking, name-calling, or humiliating comments. Indirect or relational bullying refers to less visible forms of aggression, such as social exclusion, gossip, rumor spreading, or manipulation of peer relationships [8,9]. Newer forms of peer aggression include cyberbullying, which extends bullying into digital contexts and may involve humiliating messages, denigrating comments, rumor spreading, online exclusion, or embarrassing content sharing [2,10]. This distinction between physical, verbal, indirect, and cyber forms of bullying is relevant because these behaviors may differ in visibility, perceived severity, and the extent to which observers recognize them as situations requiring intervention [11,12].

Bystander responses refer to the emotional, cognitive, and behavioral ways in which witnesses react to a bullying episode, including the extent to which they empathize with the victim, condemn the aggressor, remain passive, defend the victim, or report the situation [13,14]. Moreover, defending victims can also be understood as a specific form of prosocial behavior, because it involves recognizing another person’s distress, evaluating the situation as requiring help, and choosing a response that may protect the victim or reduce harm [15,16]. Previous research suggests that empathy is closely related to defending behavior in bullying situations [15], while moral sensitivity moral disengagement, perceived responsibility, and self-efficacy also shape whether witnesses remain passive, support the aggressor, or intervene on behalf of the victim [17,18]. Therefore, bystander responses do not represent only behavioral reactions to aggression, but can also reflect broader emotional and socio-moral processes that are central to prosocial action.

However, these reactions can also be linked to the different recognition of specific behaviors. Namely, more visible and direct forms of bullying tend to be recognized more easily as serious, by both students [19] and teachers [11], whereas relational or indirect forms are often perceived as less severe and elicit lower empathy and lower willingness to intervene [13].

For example, Bauman and Del Rio [11] found that teachers rated relational bullying as the least serious form of bullying, reported the least empathy for victims of relational bullying, and were less likely to intervene in such incidents. Similar patterns have also been observed in students’ appraisals, suggesting that these types of judgments may be found across different groups [19]. Moreover, witnesses’ perceptions of bullying shape their behavioral reactions to such events. Stronger emotional and physiological reactions to witnessing bullying are associated with a lower likelihood of remaining passive [20].

In contrast, cyberbullying seems to be more ambiguous, online aggression being often interpreted as joking, teasing, or banter [21]. This may blur the boundary between humor and harm determining students to characterize it as mere teasing or acceptable behavior. Despite this ambiguity related to online aggression, its impact should not be minimized, as cyberbullying has been consistently associated with negative psychological and behavioral outcomes for both victims, as well as aggressors [22,23]. These effects are similar to other forms of school aggression such as physical, verbal and indirect bullying [24].

Based on a similar reasoning, verbal bullying can also be perceived as ambiguous. Despite the fact that it is a direct, explicit type of aggression, it is also relatively common in peer interactions and may therefore be more easily normalized [11]. Insults, mocking, name-calling, or threats are clearly aggressive behaviors, but observers may sometimes perceive them as less serious than physical aggression, especially when no visible injury is involved [13]. Thus, although verbal bullying is more explicit than other forms such as indirect bullying, the absence of visible physical harm may still reduce perceived severity and weaken observers’ sense that intervention is necessary.

Indirect bullying raises a different difficulty, probably being one of the most overlooked form of school aggression by students and teachers alike [25,26]. It involves practices such as social exclusion, rumor spreading, and the manipulation of peer relationships [25]. Therefore, its harmful character may be less visible and more easily interpreted as interpersonal conflict, group preference, or as social distancing strategy [27]. Moreover, because indirect bullying often unfolds through several covert social acts rather than through a single clearly observable incident, observers may find it harder to identify intent, responsibility, and the actual harm experienced by the victim [28]. This ambiguity may reduce perceived seriousness, empathy toward the victim, and the perceived obligation to intervene or report the situation [11].

Beyond bullying type, previous research suggests that bystander responses are also influenced by other factors such as characteristics of the incident, the observer, and the broader social context. For example, witnesses’ appraisals and behavioral responses may depend on how serious they perceive the situation and how strongly they feel personally responsible to intervene [29]. Other factors shaping their reactions include empathy and personal similarity to the victim [30], perceived self-efficacy in defending others, and moral disengagement [17]. Among the victim-related characteristics, group membership is a particularly relevant factor [7].

Characteristics such as ethnicity, nationality, or immigrant background of the victim might make bullying episodes more or less relevant to the observer [7,31]. These stigma-related characteristics (i.e., ethnicity, religion, gender, sexual orientation, or disability) can shape how bullying is perceived and addressed, particularly when responses are influenced by prejudice, social distance, and discriminatory peer norms [32].

Ethnic minorities or socially devalued groups are often perceived through pre-existing stereotypes, including evaluations of lower warmth, lower competence, or greater social distance [33,34]. In the Romanian context, the Roma and the Hungarian minorities are officially recognized as the two largest ethnic minorities according to the 2021 Romanian Census where 89.3% declared themselves Romanian, 6.0% Hungarian, and 3.4% as Roma [35]. Although both tend to be perceived in a rather negative than a positive manner, they are associated with distinct social positions and histories of intergroup tension [36]: the Hungarian minority is mostly associated with social conflicts and injustice, while the Roma minority appears to involve stronger associations with low social status and disgust-related stereotypes [37]. Therefore, these two groups were selected because they are both socially salient ethnic minorities in Romania, but also because they differ in the type of stereotypes and intergroup meanings usually associated with them. Including both groups allowed us to examine whether bystander responses would be shaped by victim ethnicity in general, or whether they might differ depending on the specific social meanings attached to different minority groups in the local context.

In a bullying situation, these negative stereotypes may influence how observers relate to the victim and interpret the aggression itself. In line with the just-world theory, people are motivated to believe that the world is fair and that individuals generally get what they deserve [38]. When applied to victimization, this belief may lead observers to attribute part of the responsibility to the victim or to perceive the victim’s suffering as less unfair [39]. Therefore, when the victim belongs to an ethnic outgroup or to a socially marginalized group, prejudicial beliefs may translate into lower empathy, weaker condemnation of the aggressor, and a reduced tendency to engage in protective behaviors, such as defending the victim or reporting the incident [31]. At the same time, this may not necessarily imply open hostility toward outgroup victims. It might also stem from a stronger concern and protection for ingroup members rather than explicit rejection of outgroup members [40].

The negative consequences of passive bystanding do not remain limited to the victim. They may also extend to the peer-group level, by signaling tacit acceptance of the aggression and contributing to a social context in which bullying is tolerated, reinforced, or repeated [8]. In this sense, bystanders are not neutral observers: their reactions can either reinforce the aggressor, signal tacit acceptance of the behavior, or help interrupt the bullying episode [41,42]. Moreover, passivity may also be psychologically costly for bystanders themselves.

Failing to intervene, especially when recognizing the victim’s suffering can involve moral tension and negative emotion [17]. Prior research on bystander behavior in bullying situations has linked moral distress to defending, passive bystanding, and pro-bullying responses [43]. This is also consistent with moral injury theory, which argues that psychological distress may follow from perpetrating, witnessing, failing to prevent, or remaining silent in situations that violate one’s moral beliefs [44]. Empirical findings support that by showing that witnessing bullying is associated with higher anxiety and depressive symptoms [5,45], while bullying involvement is linked to psychological difficulties [46].

Although previous research has examined bystander responses and appraisals of different bullying situations, studies using vigniette-based or experimental approaches remain relatively fewer, especially when compared with studies based on retrospective self-reports of bullying involvement. This distinction is relevant because hypothetical scenarios allow participants to evaluate the same standardized situations, while the manipulation of specific cues, such as bullying type or victim ethnicity, makes it possible to examine how these features shape observers’ reactions [47]. At the same time, asking participants how they would react in a hypothetical situation may be less personally threatening than asking them to report only their own past behavior [48] and may reduce social desirability concerns, especially in a between subject design [49]. Moreover, even fewer studies combine hypothetical scenario-based appraisals with behavioral intentions and indicators of participants’ own previous bullying experiences. Therefore, assessing these factors within the same design, while also taking into account psychological distress, may offer a more integrative understanding of how observers evaluate bullying situations and how these appraisals relate to behavioral intentions. It may also clarify whether psychological distress is associated both with responses to hypothetical bullying scenarios and with participants’ own previous bullying experiences.

In line with these observations, the present study had three aims. First, we aimed to investigate the effects of bullying type on empathic reactions, appraisal of the aggressor’s behavior, and bystander behavioral intentions in four hypothetical scenarios describing physical, verbal, indirect, and cyberbullying. Second, we aimed to examine whether victim ethnicity, alongside bullying type, influenced witnesses’ emotional reactions, behavioral intentions, and judgments of aggressive behavior. Lastly, we aimed to explore whether psychological distress, reflected in symptoms of depression, anxiety, and stress, was associated with previous bullying experiences as a victim, aggressor, or witness, as well as with participants’ responses to the hypothetical bullying scenarios. Concretely, our study aimed to answer three main research questions. Do participants’ empathic reactions, appraisals of the aggressor’s behavior, and bystander intentions differ depending on the type of bullying described in the vignette? Does the victim’s ethnicity influence participants’ emotional, evaluative, and behavioral responses to bullying situations?

Are previous bullying experiences and scenario-based bystander responses associated with psychological distress?

## 2. Materials and Methods

### 2.1. Participants

The participants were adults recruited through invitations sent to university students (bachelor and master’s), who were also encouraged to share the survey further with other students (i.e., friends or acquaintances). Initially, a total of 619 responses were recorded through the SoSci Survey online survey platform (Version 3.8.08; SoSci Survey GmbH, Munich, Germany). Before running the analyses, we screened the data and excluded participants based on data quality criteria, such as incomplete responses, unusually fast response times based on SoSci Survey’s Relative Speed Index (i.e., one SD above the sample mean) and time spent per vignette pages (i.e., lower than 30 s), which suggested that the questionnaire had not been completed carefully. After these exclusions, the final sample included 477 participants aged between 18 and 52 years old (M age = 21.77, *SD* = 4.18), of whom 309 were women, 165 were men, and 3 identified as other genders. All participants completed the questionnaire voluntarily and anonymously. Young university students were targeted because the vignettes described peer-group situations in an educational context and because this population is still relatively close to school and university peer dynamics. Moreover, using an adult sample allowed participants to provide informed consent independently and to evaluate morally and socially sensitive scenarios concerning ethnicity and bullying. No financial incentives were offered but participants received course credits for participation and for inviting other participants to fill in the questionnaire. All participants were informed about the purpose of the study and provided their consent before taking part. The research was conducted in accordance with the American Psychological Association (APA) ethical standards in the treatment of human research individuals. Additionally, this work adheres to the provisions of the Declaration of Helsinki from 1995, revised at Edinburgh in 2000.

### 2.2. Design and Procedure

This study employed a mixed 3 × 4 design, with victim ethnicity as a between-subjects factor and type of bullying as a within-subjects factor. Participants were randomly assigned to one of three experimental conditions in which the ethnicity of the victim was manipulated (i.e., Romanian, Roma, or Hungarian). The questionnaire was created and administered online using SoSci Survey. After providing informed consent, participants completed the questionnaire anonymously and voluntarily. Each participant then read each of the four short bullying vignettes describing physical bullying, verbal bullying, indirect bullying, and cyberbullying (see Supplementary Material for full vignette texts and items). To reduce possible order effects, the four scenarios were presented in randomized order. After each vignette, participants answered six questions regarding their appraisals of the situation and their likely bystander reactions. Upon completing the vignette task, participants also filled in the DASS-21 scale and three items regarding past bullying experiences. These scales were presented in the same order.

### 2.3. Materials and Measures

#### 2.3.1. Bullying Appraisals

Bullying appraisals were based on participants’ judgments of four short vignettes describing four bullying forms: physical, verbal, indirect and cyber-bullying. The scenarios ranged from 80 to 131 words and described typical bullying behaviors associated with each form: intentional tripping/pushing in the physical bullying vignette, posting humiliating comments on social media in the cyberbullying vignette, excluding a colleague from a WhatsApp group in the indirect bullying vignette, and making a public humiliating comment about a colleague while the person was physically present in the verbal bullying vignette. In each of the bullying scenarios only the victim’s ethnicity was varied, the rest of the text remained identical. An example of the cyberbullying scenario translated into English is provided below (see Supplementary Material for full vignette texts and items).

*Vignette for cyberbullying*

*Imagine that you are scrolling through the feed of a social networking platform, for example Instagram or TikTok, and you see a post by one of your classmates. In the photo, the classmate is wearing a traditional [Roma/Hungarian/Romanian] costume and has written in the caption:*

*“Proud to be part of the [Roma/Hungarian/Romanian] community.”*

*Shortly after the post, another classmate comments:*

*“How can you post something so cringe?”*

*The comment is followed by other mocking reactions and laughing emojis from several classmates.*

For each of the four situations, participants evaluated on a 1 to 5 scale (1 = not at all sorry/totally acceptable, 5 = very sorry/totally unacceptable) their empathic reaction towards the victim (e.g., “How sorry are you for the person that was pushed?”) and their appraisal of the aggressor’s behavior (e.g., To what degree do you consider the behavior described as being unacceptable?). These two questions were treated as separate vignette-level outcomes and were analyzed individually.

#### 2.3.2. Bystander Intentions

For each of the four bullying scenarios, participants answered 4 questions related to their probable behavioral reactions in the imagined situation. On a 1 to 5 scale (1 = not at all likely and 5 very likely) participants indicated how likely they would be to engage in four possible bystander responses if they witnessed a similar situation: not getting involved because they considered it none of their business (e.g., “Don’t get involved because it is not my business”), join their colleagues and actively participate in the bullying (e.g., “Also laugh at the victim together with your colleagues”), defending the person who had been aggressed (e.g., “Intervene and defend the victim”), and reporting the situation to a superior or authority figure (e.g., “Report the incident to a professor or other authority figure”). Because the questions were adapted to each particular scenario, the completed set of questions is presented in Supplementary Material. These items were treated as distinct facets of bystander behaviors rather than a unitary scale.

#### 2.3.3. Psychological Distress

Psychological distress was assessed using the 21-item version of the Depression Anxiety Stress Scales (DASS-21) [50]. The instrument includes three subscales measuring depression, anxiety, and stress. Participants rated all items using the standard 1 to 4 Likert scale response format (1 = did not apply to me at all, 2 = applied to me to some degree or some of the time, 3 = applied to me to a considerable degree or a good part of time, 4 = applied to me very much or most of the time). In the analyses, the three DASS subscales were examined separately and had satisfactory internal consistency coefficients (*α* anxiety = 0.86, *α* depression = 0.89, *α* stress = 0.86). The overall internal consistency coefficient was also acceptable (*α* = 0.95).

#### 2.3.4. Past Bullying Experiences

Past bullying experiences in three different roles (i.e., as victim, witness, or aggressor) were reported by participants. Each role was assessed through a single item (e.g., “Did you experience in the past bullying as a victim/witness/aggressor”) rated on a 5-point frequency scale ranging from never to very often. Each response was analyzed individually.

## 3. Results

### 3.1. Correlation Analysis

Table 1 presents the descriptive statistics and the Spearman correlation analyses among the variables. For the bullying appraisals and bystander intentions we aggregated the responses across the scenarios. The total DASS score was significantly associated with emphatic reaction (*r* = 0.11, *p* = 0.01), appraisal of the aggressor’s behavior (*r* = 0.11, *p* = 0.01) and past experiences as victim (*r* = 0.40, *p* < 0.001) and witness (*r* = 0.27, *p* < 0.001). We found similar results for stress and anxiety, with the latter also being related to willingness to report the situations (*r* = 0.12, *p* = 0.006). Depression was significantly associated with the willingness not to get involved (*r* = 0.11, *p* = 0.01) and the past experiences as victim (*r* = 0.39, *p* < 0.001), witness (*r* = 0.26, *p* < 0.001) and aggressor (*r* = 0.10, *p* = 0.02).

### 3.2. Effects of the Experimental Conditions on Moral Appraisals, Emotional Reactions and Bystanding Behavioral Intention

#### 3.2.1. Empathic Reactions

**Figure 1:**
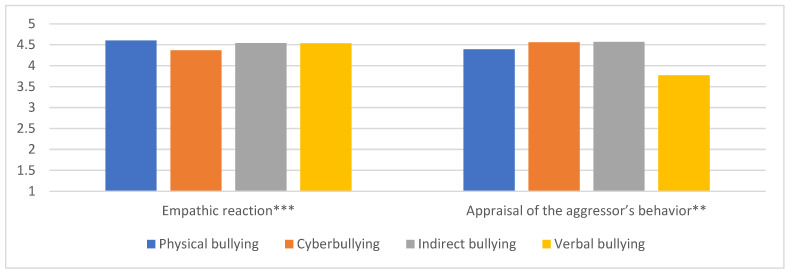
The main effect of type of bullying on bullying appraisals. *Note.* Higher scores indicate stronger empathic reaction and higher perceived unacceptability. *** *p* < 0.001; ** *p* < 0.01.

We conducted a series of Mixed ANOVAS where the victim’s ethnicity was used as a between-subjects factor and the type of bullying as a within-subjects factor. Bonferroni adjustments were applied for multiple comparisons. Gender was controlled in each analysis. For empathic reaction, Mauchly’s Test of Sphericity was significant (*p* < 0.001) and the epsilon value was greater than 0.75, the Huynh-Feldt correction was applied. The victim’s ethnicity had no significant main effect (*F*(2, 473) = 1.78, *p* = 0.173, *η_p_*^2^ = 0.007). We found a significant main effect for the type of bullying (*F*(3, 473) = 6.43, *p* < 0.001, *η_p_*^2^ = 0.013). The scores in the cyberbullying condition (M = 4.37, SE = 0.04) were significantly lower compared to physical bullying (M = 4.60, SE = 0.03), verbal bullying (M = 4.53, SE = 0.03), and indirect bullying (M = 4.53, SE = 0.03) conditions (all *p* values < 0.001). No other comparison was significant (see Figure 1). Finally, there was no significant interaction effect of victim’s ethnicity and type of bullying on empathic reaction (*F*(6, 473) = 1.06, *p* = 0.38, *η_p_*^2^ = 0.004).

#### 3.2.2. Appraisal of the Aggressor’s Behavior

In this case, higher scores indicate that the participants considered the event more unacceptable. The Mauchly’s Test of Sphericity was significant (*p* < 0.001) and the epsilon value was lower than 0.75, the Greenhouse-Geisser correction was applied. We found no significant main effect of the victim’s ethnicity (F(2, 473) = 0.75, *p* = 0.47, *η_p_*^2^ = 0.003). However, there was a significant main effect of the type of bullying (F(3, 473) = 6.02, *p* = 0.002, *η_p_*^2^ = 0.013). The scores from the verbal bullying condition (M = 3.77, SE = 0.02) were significantly lower compared to the physical bullying (M = 4.39, SE = 0.06), cyberbullying (M = 4.56, SE = 0.03) and indirect bullying (M = 4.57, SE = 0.03) conditions (all *p* values < 0.001). Also, the participants recorded significant lower scores in the physical bullying condition than the indirect bullying one (*p* = 0.047) (see Figure 1). Still, the interaction effect on the appraisal of the aggressor’s behavior was not significant (F(6, 473) = 2.01, *p* = 0.073, *η_p_*^2^ = 0.009).

#### 3.2.3. Not Getting Involved

The Mauchly’s Test of Sphericity was not significant (*p* = 0.051). We found non-significant main effects for either the victim’s ethnicity (F(2, 473) = 1.78, *p* = 0.69, *η_p_*^2^ = 0.007) and the type of bullying (F(3, 473) = 2.26, *p* = 0.10, *η_p_*^2^ = 0.004). Similarly, there was no interaction effect (F(6, 473) = 0.80, *p* = 0.56, *η_p_*^2^ = 0.003).

#### 3.2.4. Joining and Actively Participating in the Bullying

**Figure 2:**
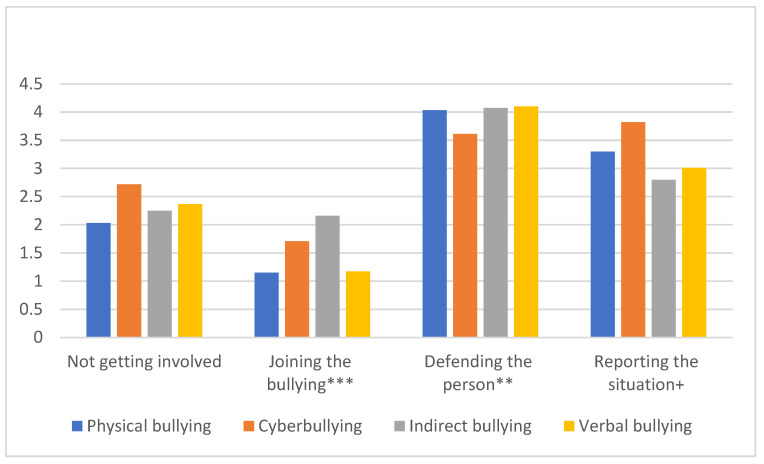
The main effect of type of bullying on bystander intentions. *Note.* Higher scores indicate greater willingness to engage in each bystander response. Error bars represent standard errors. *** *p* < 0.001; ** *p* < 0.01; + *p* < 0.06.

The Mauchly’s Test of Sphericity was significant (*p* < 0.001) and the epsilon value was lower than 0.75, the Greenhouse-Geisser correction was applied. The victim’s ethnicity had no significant main effect (F(2, 473) = 0.68, *p* = 0.50, *η_p_*^2^ = 0.003). We found a significant main effect for the type of bullying (F(3, 473) = 14.39, *p* < 0.001, *η_p_*^2^ = 0.030). The scores in the physical bullying condition (M = 1.15, SE = 0.02) were significantly lower compared to those in the cyberbullying (M = 1.71, SE = 0.05) and indirect bullying (M = 2.16, SE = 0.05) conditions. In the cyberbullying condition, the scores were higher than in the verbal bullying condition (M = 1.17, SE = 0.02), but lower compared to the indirect bullying condition. The scores from the verbal bullying condition were lower than those from the indirect bullying condition (all *p* values < 0.001) (see Figure 2). Finally, there was no significant interaction effect of victim’s ethnicity and type of bullying on joining and actively participate in the bullying (F(6, 473) = 0.49, *p* = 0.74, *η_p_*^2^ = 0.002).

#### 3.2.5. Defending the Person Who Had Been Aggressed

The Mauchly’s Test of Sphericity was significant (*p* < 0.001) and the epsilon value was greater than 0.75, when the Huynh-Feldt correction was applied. We found no significant main effect of the victim’s ethnicity (F(2, 473) = 1.26, *p* = 0.28, *η_p_*^2^ = 0.005), but there was a significant main effect of the type of bullying (F(3, 473) = 5.25, *p* = 0.002, *η_p_*^2^ = 0.011). The scores from the cyberbullying condition (M = 3.61, SE = 0.05) were significantly lower than those from the physical (M = 4.03, SE = 0.04), indirect (M = 4.07, SE = 0.05) and verbal bullying (M = 4.10, SE = 0.04) conditions (all *p* values < 0.001) (see Figure 2). There was no significant interaction effect (F(6, 473) = 0.55, *p* = 0.75, *η_p_*^2^ = 0.002).

#### 3.2.6. Reporting the Situation

**Figure 3:**
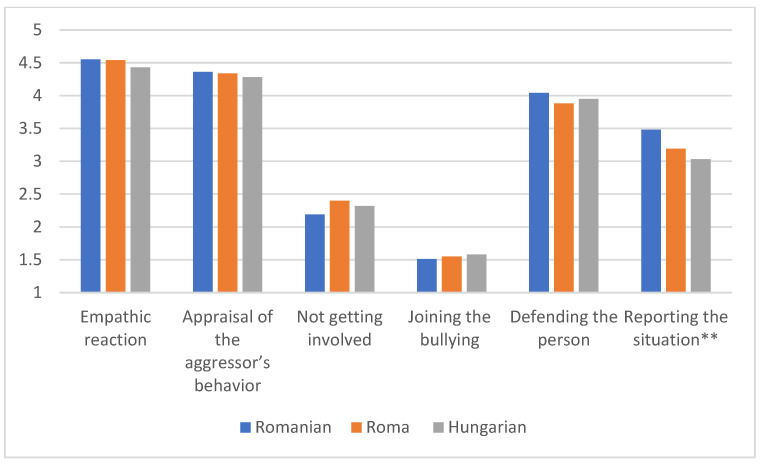
The main effect of victim’s ethnicity on bullying appraisals and bystander intentions. *Note.* ** *p* < 0.01.

The Mauchly’s Test of Sphericity was significant (*p* < 0.001) and the epsilon value was greater than 0.75, the Huynh-Feldt correction was applied. There was a main effect of the victim’s ethnicity (F(2, 473) = 6.22, *p* = 0.002, *η_p_*^2^ = 0.026). The scores were higher when the victim was Romanian (M = 3.48, SE = 0.09) than when the victim was Hungarian (M = 3.03, SE = 0.09) (*p* = 0.002). The comparisons involving the Roma victim (M = 3.19, SE = 0.09) were not significant (see Figure 3). We also found a marginally significant main effect on the type of bullying on the participants’ willingness to report the situation (F(3, 473) = 2.67, *p* = 0.054, *η_p_*^2^ = 0.006). There were significantly higher scores in the cyberbullying condition (M = 3.82, SE = 0.06) than in all the other conditions. Also, the scores from the indirect bullying condition (M = 2.89, SE = 0.06) were significantly lower than in all the other conditions. In the physical bullying condition (M = 3.30, SE = 0.06), the scores were higher than in the verbal bullying condition (M = 3.01, SE = 0.06) (all *p* values < 0.001) (see Figure 2). There was no significant interaction effect (F(6, 473) = 1.35, *p* = 0.21, *η_p_*^2^ = 0.006).

## 4. Discussion

The present study examined participants’ emotional, cognitive, and behavioral appraisals of different types of hypothetical bullying scenarios (i.e., verbal, physical, indirect and cyberbullying). It further investigated whether the victim’s ethnicity (i.e., Romanian, Roma or Hungarian) influenced these evaluations and if specific response patterns and past bullying experiences were related to psychological distress. Overall, bullying type emerged as the most consistent factor shaping participants’ responses, although the pattern differed across outcomes: cyberbullying elicited lower empathy and lower defending but higher reporting, verbal bullying was judged as less unacceptable than the other forms, and indirect bullying was associated with the highest willingness to join and the lowest willingness to report.

In contrast, victim ethnicity had a more limited effect, observed only in relation to the Hungarian minority and only for reporting intentions. Psychological distress was mainly associated with previous bullying experiences, especially victimization and witnessing. Its associations with bystander responses were smaller and more specific: depression was related to greater willingness to remain uninvolved, whereas anxiety was related to greater willingness to report.

Cyberbullying elicited lower empathic reactions compared to physical, verbal, and indirect bullying. One possible interpretation is related to the specific nature of online aggression, where the victim’s suffering is mediated through a screen and may therefore appear less immediate or less emotionally accessible to observers [51,52].

Another possible mechanism explaining the lower empathy in cyberbullying may be related to victim-blaming processes, as individuals who are visible online, post personal content, or disclose information about themselves may be implicitly perceived as partly responsible for their victimization [53,54]. At the same time, in digital contexts, cyberbullying is often reinterpreted as a form of trolling, with users perceiving aggressive behavior as more permissible online than in offline interactions [55]. However, although these explanations are plausible and generally in line with the literature, they should be regarded as speculative, since our study did not directly assess the way participants interpret or perceive online teasing, or the way they perceive the victim.

This finding is also consistent with broader evidence showing that empathy is associated with bystander helping behavior in cyberbullying, partly through moral judgment and perceived online self-efficacy [16]. Thus, lower empathy in the cyberbullying vignette may be important not only as an emotional reaction, but also because it could be related to the way in which the event itself is interpreted and the perceived responsibility to help.

Verbal bullying showed a different profile: participants appraised it as less unacceptable than physical, indirect, and cyberbullying, suggesting that this type of aggression may be more easily normalized in peer interactions. Insults, mocking, name-calling, or teasing are direct and explicit aggressive behaviors [56], but because they are relatively frequent in student interactions [57], observers may interpret them as rude or inappropriate communication rather than as a serious bullying episode. This interpretation is consistent with previous research suggesting that bullying situations involving visible harm or more concrete threats tend to be considered more serious compared to situations in which bullying consists of malicious verbal remarks [13,58]. Thus, the lower condemnation of verbal bullying in the present study may reflect a lower perceived severity of the transgression rather than a failure to recognize verbal aggression as problematic.

Participants’ behavioral intentions also varied across conditions. For example, indirect bullying elicited the highest willingness to join the aggression and the lowest willingness to report the incident. This pattern may reflect the specific nature of indirect aggression, which usually consist of covert and socially embedded behaviors, such as exclusion, gossip, rumor spreading, or peer relationship manipulation [25,27]. For example, going along with exclusion may be perceived as a minor social act or even as an omission rather than as direct participation in aggression, although it still contributes to the victim’s harm [59,60]. This interpretation is also consistent with research showing that harmful actions carried out indirectly are judged less negatively compared to direct harmful actions [61]. Finally, these characteristics may make indirect bullying harder to report, because the aggression is less visible, more difficult to document, and more easily interpreted as interpersonal conflict or simply group preference [26].

Another interesting pattern of results concerning behavioral intentions emerged in cyberbullying. Although participants reported lower willingness to actively defend the victim in the cyberbullying condition, they also reported higher willingness to report the incident. In addition, willingness to join the aggression was higher in cyberbullying than in physical and verbal bullying, although lower than in indirect bullying. This may be explained by the fact that, in online contexts, joining the aggression can involve behaviors that appear small or insignificant, such as liking, sharing, commenting, or laughing at humiliating content [62]. Because these actions are screen mediated and may occur without direct confrontation with the victim, they may feel less aggressive than overt participation in face-to-face bullying [51,52]. At the same time, cyberbullying can be witnessed by larger audiences, both during the incident and after the content has been posted or shared. Such contexts may create conditions in which bystander processes such as diffusion of responsibility, evaluation apprehension, or pluralistic ignorance become relevant [63,64]. Moreover, defending the victim may feel publicly exposed and socially risky, especially when many others are present but remain passive [65]. Reporting, in contrast, may appear as a more available and less directly confrontational response, as social networking platforms provide options such as reporting or blocking abusive content [66]. However, because these processes were not directly measured in the present study, they should be understood as possible explanations rather than confirmed mechanisms.

Finally, participants reported the lowest willingness to join physical aggression while also stating higher willingness to report physical bullying compared to other forms of aggression (i.e., verbal aggression). This pattern is consistent with the idea that physical bullying is one of the most visible and direct forms of aggression, involving bodily contact, threat, or possible injury, which makes the transgression harder to minimize or reinterpret as ordinary peer disagreement [13]. Previous research also found that physical bullying is generally perceived as more serious and more likely to require adult or institutional intervention than less visible or less concrete forms of bullying [11,58]. Therefore, actively joining physical bullying may be perceived as a more explicit and morally costly form of participation, whereas reporting it may feel more legitimate because the harm is more visible, documentable, and institutionally actionable.

Regarding victim ethnicity, the findings were more limited. Victim ethnicity did not influence empathic reactions, appraisal of the aggressor’s behavior, willingness to remain uninvolved, joining, or defending. The only significant effect emerged for reporting intentions: participants were less willing to report the situation when the victim was Hungarian than when the victim was Romanian, but there were no significant differences for the Roma minority. This result might be interpreted in relation to previous research showing that, in the Romanian context, the Hungarian and Roma minorities are associated with different stereotype profiles. Namely, the Hungarian minority appears to be more closely linked to intergroup conflict and perceived injustice, whereas the Roma minority involves stronger associations with low social status and disgust-related stereotypes [37]. However, because this was the only significant ethnicity-related effect, and because participants’ own ethnic identity, prejudice, and intergroup contact were not directly assessed, this interpretation should remain cautious.

Overall, the limited ethnicity effects may also reflect methodological characteristics of the manipulation and social desirability concerns. Since victim ethnicity was manipulated only through the textual description of the victim, without additional cues (i.e., names, images) it might have been less salient compared to a more obvious visual manipulation. At the same time, the topic of ethnicity, prejudice, and prosocial responses to bullying is socially sensitive [67]. Consequently, participants may have avoided expressing acceptance of bullying behaviors, irrespective of who the target was simply because such behaviors are generally not tolerated.

The third aim of the study was to explore whether previous bullying experiences and bystander responses were associated with psychological distress (i.e., depression, anxiety, and stress). Overall, the strongest associations concerned participants’ past bullying experiences: previous victimization and witnessing were consistently related to all three facets of psychological distress. This pattern is consistent with previous research showing that bullying victimization is associated with later mental health difficulties, including depression and anxiety [46] and with studies indicating that witnessing bullying is associated with distress [5,45]. Furthermore, past experience as an aggressor was also associated with higher depression, a finding consistent with meta-analytic evidence showing that bullying perpetration is also associated with depressive symptoms, even if the association is generally weaker and more complex than in the case of victimization [68].

Psychological distress was only selectively related to scenario-based bystander intentions: higher depression was associated with greater willingness to remain uninvolved, whereas higher anxiety was associated with greater willingness to report the situation. The fact that these associations were smaller than those observed for past bullying experiences is not surprising, given that participants responded to hypothetical scenarios rather than reporting actual behavior. Although vignette-based designs allow standardized situations and controlled manipulations, they assess imagined responses and may not fully capture how individuals behave in real-life situations [47]. At the same time, meta-analytic work showing that intentions are among the closest predictors of later behavior, support the relevance of these results [69,70]. Therefore, although the associations between psychological distress and scenario-based responses were smaller, they complete the broader pattern observed in the present study: participants’ previous bullying experiences were more strongly linked to psychological distress, whereas their imagined bystander responses showed weaker but still meaningful association with specific forms of distress.

### 4.1. Limitations

Several limitations should be considered when interpreting these findings. First, the limited ethnicity effects may reflect not only the absence of strong ethnicity-based differences in participants’ responses, but also the relatively subtle nature of the manipulation. Victim ethnicity was introduced only through the textual description of the victim, without additional cues such as images, names, accents, or contextual details that might have made group membership more salient. Although between-subjects manipulations may reduce the likelihood that participants infer the scope of the study [49], they do not eliminate socially desirability response. Given the social salience of the Roma, and Hungarian minorities in the local context [36], some participants may still have assumed the aim of the study and responded in a normatively desired manner. Moreover, even if participants did not directly assume the aim of the study, social desirability concerns still might have influenced the results because participants might have avoided expressing lower empathy, weaker condemnation, or reduced willingness to help minority victims because such responses could be perceived as prejudiced or morally inappropriate [67]. Future studies could use richer and more immersive manipulations, such as pictures, videos, or VR technologies to increase the salience of the manipulation. In line with these observations it should be noted that, apart from the experimental manipulation, the study also discussed the associations involving psychological distress and previous bullying experiences based on cross-sectional self-report data. Therefore, these associations should not be interpreted causally. Moreover, the bystander responses assessed in the vignette task reflected behavioral intentions rather than actual behavior in real bullying situations. Future studies should combine experimental vignette designs with longitudinal or observational approaches in order to better examine how distress, previous experiences, and real bystander behavior are related over time.

Second, the study was administered online and completed independently by participants, which limited the degree of control over the testing context. Although this format allowed the recruitment of a larger sample and may have made participation more comfortable, it also means that some participants may have completed the questionnaire in distracting environments or with varying levels of attention. Future research could replicate the design in more controlled settings or include additional attention and manipulation checks.

Thirdly, previous bullying experiences were assessed through only three single items, corresponding to victim, aggressor, and witness roles. Although this allowed a relatively brief questionnaire, thus avoiding participants’ fatigue, it did not capture the complexity, frequency, duration, severity, or developmental timing of these experiences. Future studies should use more detailed scales of bullying involvement and should also include measures of social desirability, especially given the sensitive nature of ethnicity, prejudice, and bystander responses in bullying situations.

In addition, the study examined responses to bullying scenarios in a university context and used an adult sample, mostly composed of young college-aged participants. Therefore, although the scenarios described bullying behaviors that can also occur in educational settings, the generalization of these results to actual school settings and school-aged adolescents is limited. Moreover, because participants were recruited through convenience and snowball sampling, the sample cannot be considered representative of the broader student or adolescent population. Future studies should test whether similar mechanisms can also be observed among children and adolescents in real peer interactions.

### 4.2. Implications for Prevention and Intervention

The present findings also have practical implications for prevention and intervention. First, they suggest that anti-bullying programs should not treat bullying as a single, homogeneous category. While physical bullying may be more easily recognized as serious, other forms such as verbal, indirect, and cyber forms of aggression might be overlooked because they often imply subtle mechanisms that may be more easily normalized. Therefore, prevention efforts should explicitly address the everyday language through which harm is minimized, such as “it was only a joke,” “you are too sensitive,” “this is normal peer teasing,” or “it was just a comment/like.” Such messages may reduce empathy for the victim and make bystanders less likely to recognize their own role in maintaining the aggression.

Second, interventions should help students distinguish between different forms of bystander involvement and provide concrete, safe alternatives for action. While direct defending might be avoided by most students because of concerns for their own safety [65], indirect forms of support, such as comforting the victim, refusing to join gossip or exclusion, documenting harmful online content, anonymously reporting the incident, or seeking help from an adult might be preferred by students. Finally, the findings underline the importance of creating inclusive school and online environments in which victims and witnesses feel supported, and in which reporting is encouraged regardless of the victim’s group membership. Moreover, educational intervention that teach students what is safe to do, how to help directly, indirectly and anonymously, raising awareness about indirect participation in bullying might show individuals that there is more than one way to take a stand against bullying [71]. In this sense, prevention programs should not only inform students about bullying types, but also strengthen the emotional and socio-moral competencies that support prosocial bystander behavior [15,18]. This may include activities focused on perspective-taking, empathic concern, recognition of subtle harm, and practical and safe ways of defending victims. Evidence from bystander-focused interventions suggests that such programs can increase bystander intervention, especially when they provide concrete skills and increase students’ confidence that they can act safely and effectively [71,72]. In cyberbullying contexts, interventions should also include digital self-efficacy and practical online response strategies, such as documenting harmful content, reporting it, refusing to amplify it through likes or comments, and offering private support to the victim. Moreover, because emotional intelligence and related socio-emotional competencies, such as emotion recognition, emotion understanding, and emotion regulation, have been linked to lower involvement in bullying behaviors, these components may be useful additions to bystander-oriented interventions [73]. Finally, because prosocial behavior is also shaped by cultural values and intergroup meanings [74], interventions should be culturally sensitive and should explicitly address how ethnicity, stereotypes, and perceived group membership may influence empathy, reporting, and defending.

## 5. Conclusions

Taken together, these results suggest that bystander responses to bullying are differentiated rather than uniform. Bullying type was the most consistent factor shaping participants’ reactions, but its effects varied across emotional, evaluative, and behavioral outcomes. Physical bullying emerged as the form least likely to be joined, indirect bullying was judged as the most acceptable form of aggression, while cyberbullying elicited a specific profile, involving lower empathy and lower willingness to defend the victim, but higher willingness to report the incident. Victim ethnicity played a more limited role, relating only to reporting intentions and only for the Hungarian minority. Finally, psychological distress was associated with both previous bullying experiences and scenario-based responses, although relating more strongly to the former than the latter. Overall, our findings support the need to examine bullying not only as a general category of aggression, but as a set of situations that differ in visibility, perceived severity, social risk, and perceived possibilities for intervention.

## Figures and Tables

**Table 1:** Means, standard deviations and correlations among the variables.

	M	*SD*	1	2	3	4	5	6	7	8	9	10	11	12
1. DASS total	43.65	14.	-											
2. Stress	15.93	4.9	0.92 **	-										
3. Depression	13.97	5.43	0.91 **	0.77 **	-									
4. Anxiety	13.73	5.07	0.91 **	0.78 **	0.76 **	-								
5. Empathic reaction	18.04	2.63	0.11 *	0.12 **	0.07	0.12 **	-							
6. Appraisal of the aggressor’s behavior	17.31	2.28	0.11 *	0.12 **	0.06	0.12 **	0.52 **	-						
7. Not getting involved	9.24	3.95	0.08	0.06	0.11 *	0.02	−0.31 **	−0.22 **	-					
8. Joining the bullying	6.20	2.33	0.05	0.03	0.08	0.03	−0.38 **	−0.27 **	0.50 **	-				
9. Defending the person	15.83	3.53	−0.03	−0.02	−0.06	−0.01	0.37 **	0.31 **	−0.61 **	−0.41 **	-			
10. Reporting the situation	12.96	4.64	0.05	0.05	0.01	0.12 **	0.35 **	0.28 **	−0.40 **	−0.30 **	0.53 **	-		
11. Past experience as victim	2.69	1.24	0.40 **	0.37 **	0.39 **	0.34 **	0.09 *	0.08	−0.00	0.01	0.01	0.04	-	
12. Past experience as witness	3.64	1.05	0.27 **	0.24 **	0.26 **	0.22 **	0.02	0.06	0.03	0.01	0.03	0.07	0.27 **	-
13. Past experience as aggressor	1.39	0.614	0.06	0.06	0.10 *	0.02	−0.20 **	−0.03	0.13 **	0.21 **	−0.14 **	−0.12 **	0.24 **	0.21 **

*Note. N* = 477; * *p* < 0.05; ** *p* < 0.01.

## Data Availability

The de-identified data supporting the findings of this study are available on the Open Science Framework (OSF) at https://osf.io/56rv3/ (accessed on 10 July 2026).

## Supplementary Materials

The following supporting information can be downloaded at: https://www.mdpi.com/article/10.3390/healthcare14142194/s1.

## References

[1] Biswas T., Scott J.G., Munir K., Thomas H.J., Huda M.M., Hasan M.M., De Vries D.T., Baxter J., Mamun A.A. (2020). Global Variation in the Prevalence of Bullying Victimisation Amongst Adolescents: Role of Peer and Parental Supports. eClinicalMedicine.

[2] Modecki K.L., Minchin J., Harbaugh A.G., Guerra N.G., Runions K.C. (2014). Bullying Prevalence Across Contexts: A Meta-Analysis Measuring Cyber and Traditional Bullying. J. Adolesc. Health.

[3] Olweus D. (1993). Bullying at School: What We Know and What We Can Do.

[4] Labella M.H., Klein N.D., Yeboah G., Bailey C., Doane A.N., Kaminer D., Bravo A.J. (2024). Cross-Cultural Addictions Study Team Childhood Bullying Victimization, Emotion Regulation, Rumination, Distress Tolerance, and Depressive Symptoms: A Cross-National Examination Among Young Adults in Seven Countries. Aggress. Behav..

[5] Midgett A., Doumas D.M. (2019). Witnessing Bullying at School: The Association Between Being a Bystander and Anxiety and Depressive Symptoms. Sch. Ment. Health.

[6] Prignitz M., Banaschewski T., Bokde A.L.W., Desrivières S., Grigis A., Garavan H., Gowland P., Heinz A., Martinot J.-L., Paillère Martinot M.-L. (2023). The Role of Empathy in Alcohol Use of Bullying Perpetrators and Victims: Lower Personal Empathic Distress Makes Male Perpetrators of Bullying More Vulnerable to Alcohol Use. Int. J. Environ. Res. Public Health.

[7] Hitti A., Gönültaş S., Mulvey K.L. (2023). What Motivates Adolescent Bystanders to Intervene When Immigrant Youth Are Bullied?. J. Res. Adolesc..

[8] Menesini E., Salmivalli C. (2017). Bullying in Schools: The State of Knowledge and Effective Interventions. Psychol. Health Med..

[9] Shetgiri R. (2013). Bullying and Victimization Among Children. Adv. Pediatr..

[10] Lozano-Blasco R., Cortés-Pascual A., Latorre-Martínez M.P. (2020). Being a Cybervictim and a Cyberbully—The Duality of Cyberbullying: A Meta-Analysis. Comput. Hum. Behav..

[11] Bauman S., Del Rio A. (2006). Preservice Teachers’ Responses to Bullying Scenarios: Comparing Physical, Verbal, and Relational Bullying. J. Educ. Psychol..

[12] Sticca F., Perren S. (2013). Is Cyberbullying Worse than Traditional Bullying? Examining the Differential Roles of Medium, Publicity, and Anonymity for the Perceived Severity of Bullying. J. Youth Adolesc..

[13] Jungert T., Berjot S., Hong J.S., Thornberg R. (2025). Classroom Bullying: Understanding Bystander Reactions Across Different Bullying Types. J. Sch. Violence.

[14] Salmivalli C., Karhunen J., Lagerspetz K.M.J. (1996). How Do the Victims Respond to Bullying?. Aggress. Behav..

[15] Deng X., Yang J., Wu Y. (2021). Adolescent Empathy Influences Bystander Defending in School Bullying: A Three-Level Meta-Analysis. Front. Psychol..

[16] Hu Y., Zhang T., Shi H., Fan C. (2023). Empathy and Bystander Helping Behavior in Cyberbullying Among Adolescents: The Mediating Role of Internet Moral Judgment and the Moderating Role of Internet Self-Efficacy. Front. Psychol..

[17] Thornberg R., Jungert T. (2013). Bystander Behavior in Bullying Situations: Basic Moral Sensitivity, Moral Disengagement and Defender Self-efficacy. J. Adolesc..

[18] Sjögren B., Thornberg R., Kim J., Hong J.S., Kloo M. (2024). Basic Moral Sensitivity, Moral Disengagement, and Defender Self-Efficacy as Predictors of Students’ Self-Reported Bystander Behaviors over a School Year: A Growth Curve Analysis. Front. Psychol..

[19] Varjas K., Henrich C.C., Meyers J. (2009). Urban Middle School Students’ Perceptions of Bullying, Cyberbullying, and School Safety. J. Sch. Violence.

[20] Barhight L.R., Hubbard J.A., Hyde C.T. (2013). Children’s Physiological and Emotional Reactions to Witnessing Bullying Predict Bystander Intervention. Child Dev..

[21] Steer O.L., Betts L.R., Baguley T., Binder J.F. (2020). “I Feel like Everyone Does It”—Adolescents’ Perceptions and Awareness of the Association Between Humour, Banter, and Cyberbullying. Comput. Hum. Behav..

[22] Estévez E., Estévez J.F., Segura L., Suárez C. (2019). The Influence of Bullying and Cyberbullying in the Psychological Adjustment of Victims and Aggressors in Adolescence. Int. J. Environ. Res. Public Health.

[23] Kowalski R.M., Giumetti G.W., Schroeder A.N., Lattanner M.R. (2014). Bullying in the Digital Age: A Critical Review and Meta-Analysis of Cyberbullying Research Among Youth. Psychol. Bull..

[24] Huang C.L., Alimu Y., Yang S.C., Kang S. (2023). What You Think Is a Joke Is Actually Cyberbullying: The Effects of Ethical Dissonance, Event Judgment and Humor Style on Cyberbullying Behavior. Comput. Hum. Behav..

[25] Crothers L.M., Kolbert J.B., Schmitt A.J., Cowley J., Perfetto K., Vafiadis A., Zawodny A. (2026). How Do We Identify Potential Perpetrators of Indirect Bullying and How Do We Help Them? A Review of the Characteristics That Are Associated with Perpetration and Can Be Targeted Through Prevention and Intervention. Front. Psychol..

[26] Dedousis-Wallace A., Shute R., Varlow M., Murrihy R., Kidman T. (2014). Predictors of Teacher Intervention in Indirect Bullying at School and Outcome of a Professional Development Presentation for Teachers. Educ. Psychol..

[27] Archer J., Coyne S.M. (2005). An Integrated Review of Indirect, Relational, and Social Aggression. Pers. Soc. Psychol. Rev..

[28] Burton B. (2010). Dramatising the Hidden Hurt: Acting against Covert Bullying by Adolescent Girls. Res. Drama Educ. J. Appl. Theatre Perform..

[29] Nickerson A.B., Mele-Taylor D. (2014). Empathetic Responsiveness, Group Norms, and Prosocial Affiliations in Bullying Roles. Sch. Psychol. Q..

[30] Pascal E. (2019). Being Similar While Judging Right and Wrong: The Effects of Personal and Situational Similarity on Moral Judgements. Int. J. Psychol..

[31] Palmer S.B., Abbott N. (2018). Bystander Responses to Bias-Based Bullying in Schools: A Developmental Intergroup Approach. Child Dev. Perspect..

[32] Earnshaw V.A., Reisner S.L., Menino D.D., Poteat V.P., Bogart L.M., Barnes T.N., Schuster M.A. (2018). Stigma-Based Bullying Interventions: A Systematic Review. Dev. Rev..

[33] Fiske S.T., Cuddy A.J.C., Glick P., Xu J. (2002). A Model of (Often Mixed) Stereotype Content: Competence and Warmth Respectively Follow from Perceived Status and Competition. J. Pers. Soc. Psychol..

[34] Paolini S., Harwood J., Logatchova A., Rubin M., Mackiewicz M. (2021). Emotions in Intergroup Contact: Incidental and Integral Emotions’ Effects on Interethnic Bias Are Moderated by Emotion Applicability and Subjective Agency. Front. Psychol..

[35] Institutul Naţional de Statistică (2023). Recensământul Populației Și Locuințelor, Runda 2021 Date Provizorii În Profil Teritorial.

[36] Cernat V. (2019). Interminority Contact and Solidarity Under Conflict: Evidence from the Hungarian and Roma Minorities in Romania. Basic Appl. Soc. Psychol..

[37] Pascal E., Holman A.C., Miluț F.M. (2023). Emotional Relevance and Prejudice: Testing the Differentiated Effect of Incidental Disgust on Prejudice Towards Ethnic Minorities. Front. Psychol..

[38] Lerner M.J., Miller D.T. (1978). Just World Research and the Attribution Process: Looking Back and Ahead. Psychol. Bull..

[39] Hafer C.L., Bègue L. (2005). Experimental Research on Just-World Theory: Problems, Developments, and Future Challenges. Psychol. Bull..

[40] Brewer M.B. (1999). The Psychology of Prejudice: Ingroup Love and Outgroup Hate?. J. Soc. Issues.

[41] Salmivalli C. (2010). Bullying and the Peer Group: A Review. Aggress. Violent Behav..

[42] Salmivalli C., Voeten M., Poskiparta E. (2011). Bystanders Matter: Associations Between Reinforcing, Defending, and the Frequency of Bullying Behavior in Classrooms. J. Clin. Child Adolesc. Psychol..

[43] Gini G., Thornberg R., Pozzoli T. (2020). Individual Moral Disengagement and Bystander Behavior in Bullying: The Role of Moral Distress and Collective Moral Disengagement. Psychol. Violence.

[44] Litz B.T., Stein N., Delaney E., Lebowitz L., Nash W.P., Silva C., Maguen S. (2009). Moral Injury and Moral Repair in War Veterans: A Preliminary Model and Intervention Strategy. Clin. Psychol. Rev..

[45] Rivers I., Poteat V.P., Noret N., Ashurst N. (2009). Observing Bullying at School: The Mental Health Implications of Witness Status. Sch. Psychol. Q..

[46] Moore S.E., Norman R.E., Suetani S., Thomas H.J., Sly P.D., Scott J.G. (2017). Consequences of Bullying Victimization in Childhood and Adolescence: A Systematic Review and Meta-Analysis. World J. Psychiatry.

[47] Aguinis H., Bradley K.J. (2014). Best Practice Recommendations for Designing and Implementing Experimental Vignette Methodology Studies. Organ. Res. Methods.

[48] Barter C., Renold E. (2000). “I Wanna Tell You a Story”: Exploring the Application of Vignettes in Qualitative Research with Children and Young People. Int. J. Soc. Res. Methodol..

[49] Walzenbach S. (2019). Hiding Sensitive Topics by Design? An Experiment on the Reduction of Social Desirability Bias in Factorial Surveys. Surv. Res. Methods.

[50] Lovibond S.H., Lovibond P.F. (1995). Manual for the Depression Anxiety Stress Scales.

[51] Lapidot-Lefler N., Barak A. (2012). Effects of Anonymity, Invisibility, and Lack of Eye-Contact on Toxic Online Disinhibition. Comput. Hum. Behav..

[52] Barlińska J., Szuster A., Winiewski M. (2013). Cyberbullying Among Adolescent Bystanders: Role of the Communication Medium, Form of Violence, and Empathy. J. Community Appl. Soc. Psychol..

[53] Aizenkot D. (2020). Social Networking and Online Self-Disclosure as Predictors of Cyberbullying Victimization Among Children and Youth. Child. Youth Serv. Rev..

[54] Aliyu S., Salehzadeh Niksirat K., Huguenin K., Cherubini M. (2023). On the Role and Form of Personal Information Disclosure in Cyberbullying Incidents. Proc. Priv. Enhancing Technol..

[55] Hilvert-Bruce Z., Neill J.T. (2020). I’m Just Trolling: The Role of Normative Beliefs in Aggressive Behaviour in Online Gaming. Comput. Hum. Behav..

[56] Kapitanoff S., Pandey C. (2024). The Content of Verbal Bullying and Emotional Reactions Among Middle-School Students. Child Youth Care Forum.

[57] Wang J., Iannotti R.J., Nansel T.R. (2009). School Bullying Among Adolescents in the United States: Physical, Verbal, Relational, and Cyber. J. Adolesc. Health.

[58] Petrosino A., Guckenburg S., DeVoe J., Hanson T. (2010). What Characteristics of Bullying, Bullying Victims, and Schools Are Associated with Increased Reporting of Bullying to School Officials?.

[59] Royzman E.B., Baron J. (2002). The Preference for Indirect Harm. Soc. Justice Res..

[60] Baron J., Ritov I. (1994). Reference Points and Omission Bias. Organ. Behav. Hum. Decis. Process..

[61] Paharia N., Kassam K.S., Greene J.D., Bazerman M.H. (2009). Dirty Work, Clean Hands: The Moral Psychology of Indirect Agency. Organ. Behav. Hum. Decis. Process..

[62] DeSmet A., Bastiaensens S., Van Cleemput K., Poels K., Vandebosch H., Cardon G., De Bourdeaudhuij I. (2016). Deciding Whether to Look after Them, to like It, or Leave It: A Multidimensional Analysis of Predictors of Positive and Negative Bystander Behavior in Cyberbullying Among Adolescents. Comput. Hum. Behav..

[63] Latané B., Darley J.M. (1970). The Unresponsive Bystander: Why Doesn’t He Help?.

[64] Clark M., Bussey K. (2020). The Role of Self-Efficacy in Defending Cyberbullying Victims. Comput. Hum. Behav..

[65] Iotti N.O., Menin D., Jungert T. (2022). Early Adolescents’ Motivations to Defend Victims of Cyberbullying. Int. J. Environ. Res. Public Health.

[66] Khairy M., Mahmoud T.M., Abd-El-Hafeez T., Mahfouz A., Hassanien A.-E., Chang K.-C., Mincong T. (2021). User Awareness of Privacy, Reporting System and Cyberbullying on Facebook. Advanced Machine Learning Technologies and Applications.

[67] Krumpal I. (2013). Determinants of Social Desirability Bias in Sensitive Surveys: A Literature Review. Qual. Quant..

[68] Ye Z., Wu D., He X., Ma Q., Peng J., Mao G., Feng L., Tong Y. (2023). Meta-Analysis of the Relationship Between Bullying and Depressive Symptoms in Children and Adolescents. BMC Psychiatry.

[69] Ajzen I. (1991). The Theory of Planned Behavior. Organ. Behav. Hum. Decis. Process..

[70] Sheeran P. (2002). Intention—Behavior Relations: A Conceptual and Empirical Review. Eur. Rev. Soc. Psychol..

[71] Chen Q., Lin W., Wu Q., Chan K.L. (2025). The Effectiveness of Interventions on Bullying and Cyberbullying Bystander: A Meta-Analysis. Trauma Violence Abus..

[72] Polanin J.R., Espelage D.L., Pigott T.D. (2012). A Meta-Analysis of School-Based Bullying Prevention Programs’ Effects on Bystander Intervention Behavior. Sch. Psychol. Rev..

[73] Yosep I., Mardhiyah A., Kurniawan K., Maulana I. (2024). The Relationship between Emotional Intelligence and Bullying in Adolescents: A Scoping Review. OBM Neurobiol..

[74] Padilla-Walker L.M., Van Der Graaff J., Workman K., Carlo G., Branje S., Carrizales A., Gerbino M., Gülseven Z., Hawk S.T., Luengo Kanacri P. (2022). Emerging Adults’ Cultural Values, Prosocial Behaviors, and Mental Health in 14 Countries During the COVID-19 Pandemic. Int. J. Behav. Dev..

