# Supplementary material for: Standing by or Standing up? Victim Ethnicity, Bystander Responses and Psychological Distress in Different Types of Bullying Situations
Source: Healthcare (Basel). 2026 Jul 20;14(14):2194. doi: 10.3390/healthcare14142194 (PMC13411612; doi:10.3390/healthcare14142194)
Supplement: Supplementary file 1 [file healthcare-14-02194-s001.zip › healthcare-4329522-supplementary.pdf]

Supplementary Material

Vignettes and Study-Specific Questions

The study used a 3 × 4 vignette design. The between-subjects factor was the victim's ethnicity: Romanian, Roma, or Hungarian. The within-subjects factor was the type of bullying situation: physical, verbal, indirect/social exclusion, and cyberbullying. Participants were randomly assigned to one victim-ethnicity condition and then read four vignettes corresponding to the four bullying types, presented in randomized order.

The original vignettes were administered in Romanian. English translations are provided below for transparency.

A. Romanian Version

A1. Physical bullying vignette

Imaginează-ți că te afli pe holul universității și vezi unul dintre colegii tăi de grupă. Știi destul de multe lucruri despre această persoană din mediul online, de exemplu, de pe TikTok, inclusiv muzica pe care o preferă, serialele pe care le urmărește și faptul că a participat în weekendul trecut la un festival de tradiții [rome/maghiare/românești], specific comunității din care face parte.

Colegul este cunoscut în grup ca fiind deschis în a-și exprima apartenența la această comunitate.

În timpul unei pauze, un alt coleg se apropie de persoana descrisă anterior și, în timp ce trece pe hol, îi pune intenționat piedică, astfel încât persoana cade și se lovește. În momentul căderii, colegul spune:

„Hai, uită-te pe unde mergi, cei ca voi faceți întotdeauna probleme."

Unii dintre colegii aflați în apropiere încep să râdă.

A2. Cyberbullying vignette

Imaginează-ți că scrolezi pe feed-ul de pe o rețea de socializare, de exemplu, Instagram sau TikTok, și vezi o postare a unuia dintre colegii tăi de grupă. În fotografie, colegul poartă un costum tradițional [rom/maghiar/românesc] și a scris în descriere:

„Mândru/ă să fac parte din comunitatea [romă/maghiară/românească]."

La scurt timp după postare, un alt coleg comentează:

„Cum poți să postezi ceva atât de cringe (ridicol)?"

Comentariul este urmat de alte reacții batjocoritoare și emoji-uri de râs din partea unor colegi.

A3. Indirect bullying / social exclusion vignette

Imaginează-ți că studenții din grupa ta comunică de obicei printr-un grup de WhatsApp. La un moment dat, un coleg te adaugă pe un al doilea grup de WhatsApp, în care sunt toți membrii grupei, mai puțin unul dintre colegi de etnie [romă/maghiară/română].

De la seminarul de cunoaștere de săptămâna trecută, știi că persoana exclusă este deschisă, vorbăreață și dispusă să împărtășească lucruri personale despre sine, inclusiv respectul față de comunitatea [romă/maghiară/română] din care face parte.

În noul grup, un coleg scrie:

„În sfârșit suntem doar între noi și putem vorbi liber."

Mai mulți colegi reacționează pozitiv la mesaj, iar grupul este folosit în continuare pentru discuții legate de cursuri și activități comune, fără ca persoana exclusă să fie informată sau invitată.

A4. Verbal bullying vignette

Imaginează-ți că te afli în sala de curs împreună cu colegii tăi și așteptați sosirea profesorului. La un moment dat, pe grupul de WhatsApp al grupei apare un mesaj prin care sunteți anunțați că au fost afișate listele cu bursele. Mai mulți colegi încep să își verifice rezultatele pe telefoane, inclusiv un coleg despre care știi din discuțiile anterioare că ține foarte mult la tradițiile comunității [rome/maghiare/române] din care face parte.

Când unul dintre colegi îl observă, îi spune, pe un ton ironic, în fața celorlalți:

„Tu de ce te uiți pe listă? Cei ca tine nu au nicio șansă la bursă."

Mai mulți colegi încep să râdă, iar situația continuă până la sosirea profesorului.

B. English Version

B1. Physical bullying vignette

Imagine that you are in the university hallway and see one of your classmates. You know quite a few things about this person from online contexts, for example from TikTok, including the music they like, the series they watch, and the fact that last weekend they attended a festival of [Roma/Hungarian/Romanian] traditions, specific to the community they belong to.

The classmate is known in the group as being open about expressing their belonging to this community.

During a break, another classmate approaches the person described above and, while passing through the hallway, intentionally trips them, causing the person to fall and hurt themselves. As the person falls, the classmate says:

“Come on, watch where you’re going, people like you always cause problems.”

Some of the classmates nearby begin to laugh.

B2. Cyberbullying vignette

Imagine that you are scrolling through the feed of a social networking platform, for example Instagram or TikTok, and you see a post by one of your classmates. In the photo, the classmate is wearing a traditional [Roma/Hungarian/Romanian] costume and has written in the caption:

“Proud to be part of the [Roma/Hungarian/Romanian] community.”

Shortly after the post, another classmate comments:

“How can you post something so cringe?”

The comment is followed by other mocking reactions and laughing emojis from several classmates.

B3. Indirect bullying / social exclusion vignette

Imagine that students in your group usually communicate through a WhatsApp group. At one point, a classmate adds you to a second WhatsApp group that includes all members of the class group except one classmate of [Roma/Hungarian/Romanian] ethnicity.

From last week’s introductory seminar, you know that the excluded person is open, talkative, and willing to share personal things about themselves, including their respect for the [Roma/Hungarian/Romanian] community they belong to.

In the new group, one classmate writes:

“Finally, it’s just us and we can speak freely.”

Several classmates react positively to the message, and the group continues to be used for discussions related to courses and shared activities, without the excluded person being informed or invited.

B4. Verbal bullying vignette

Imagine that you are in the classroom with your classmates, waiting for the professor to arrive. At one point, a message appears in the class WhatsApp group announcing that the scholarship lists have been posted. Several classmates start checking the results on their phones, including a classmate whom you know from previous discussions to care deeply about the traditions of the [Roma/Hungarian/Romanian] community they belong to.

When one of the classmates notices them, they say ironically, in front of the others:

“Why are you checking the list? People like you have no chance of getting a scholarship.”

Several classmates start laughing, and the situation continues until the professor arrives.

C. Study-Specific Questions

After each vignette, participants answered the following questions. The wording was adapted slightly to match the specific situation described in each vignette.

1. How sorry do you feel for the person targeted in the situation?
   1 = not sorry at all; 5 = very sorry

2. To what extent do you consider the behavior described to be unacceptable?
   1 = totally acceptable; 5 = totally unacceptable

3. If you witnessed or were involved in a similar situation, how likely would you be to do each of the following?
   1 = not at all likely; 5 = very likely
   a. Not get involved, considering that it is not your business.
   b. Join or accept the situation, depending on the vignette: laugh along with the others, react positively to the mocking comments, or remain in the separate group without saying anything.
   c. Defend the targeted person.
   d. Report the incident to a teacher, university authority, or platform moderator, depending on the context.
